# Hexavalent Chromium Toxicity in the Pancreas: A Study on the Protective Effects of *Hypericum perforatum* Extract

**DOI:** 10.3390/ijms27083706

**Published:** 2026-04-21

**Authors:** Jelena Savici, Simona Marc, Oana-Maria Boldura, Catalin Cicerone Grigorescu, Cristina Paul, Cristina Văduva, Diana Brezovan

**Affiliations:** 1 Faculty of Veterinary Medicine, University of Life Sciences “King Mihai I” from Timisoara, Calea Aradului No. 119, 300645 Timisoara, Romania; jelenasavici@usvt.ro (J.S.); cristina.vaduva@usvt.ro (C.V.); dianabrezovan@usvt.ro (D.B.); 2 Faculty of Medicine, West University “Vasile Goldis”, Bd Revolutiei 94, 310025 Arad, Romania; caatalin@grigorescu.pro; 3 Faculty of Chemical Engineering, Biotechnologies and Environmental Protection, Politehnica University Timisoara, Vasile Pârvan No. 6, 300223 Timisoara, Romania; cristina.paul@upt.ro

**Keywords:** chromium VI, islets of Langerhans, *Hypericum perforatum*, *Bax/Bcl2*

## Abstract

Hexavalent chromium, a widespread heavy metal, induces apoptosis via the mitochondrial pathway through *Bax* (pro-apoptotic) and *Bcl2* (anti-apoptotic) proteins. *Hypericum perforatum*, rich in antioxidants, can neutralise free radicals. This study investigated the effects of CrVI on the pancreas and the protective role of *Hypericum perforatum*. Five groups of animals were used: control, Cr (CrVI for 3 months), CrH (CrVI + 2.5% *Hypericum perforatum* extract made from flowers, for 3 months), Cr2 (CrVI for 3 months + distilled water for 1 month), and CrH2 (CrVI for 3 months + *Hypericum perforatum* extract for 1 month). Samples were collected for histological analysis, gene expression (qRT-PCR), and blood glucose level analysis. CrVI exposure (Cr, Cr2) caused pancreatic damage: oedema, reduced islet size, endocrine cell vacuolisation, and endothelial swelling. Lesions were milder in CrH, while CrH2 resembled the control group. The *Bax/Bcl2* ratio increased under CrVI (highest in Cr2), indicating apoptosis, but decreased toward control values in CrH and CrH2. Blood glucose levels confirmed these findings. CrVI proved toxic to the endocrine pancreas, inducing structural and molecular alterations that impaired carbohydrate metabolism. Administration of *Hypericum perforatum* extract reduced these effects, confirming its antioxidant action and potential as a protective agent against CrVI-induced oxidative stress.

## 1. Introduction

The health risks posed by chromium stem from several factors: it is one of the most widespread metals in the Earth’s crust; anthropogenic sources are numerous, including the leather industry, electroplating, welding, pigment and paint production [1], which contaminate water, soil, and air through waste disposal [2]; and it was among the first metals to be classified as carcinogenic [3]. The most toxic form for animals and humans is hexavalent chromium (CrVI) due to its increased solubility and stability [2].

Over the years, it has been demonstrated that the toxic effects of chromium extend to the respiratory, digestive, cardiovascular, haematological, renal, nervous [4], and reproductive systems [5,6]. Research on the impact of chromium on endocrine glands is limited, with most studies focusing on the pituitary [7,8], thyroid [9,10,11,12,13,14], and adrenal glands [15], while data on its effects on the endocrine pancreas remain scarce [16,17,18].

The toxicity of hexavalent chromium arises from its intracellular reduction to trivalent chromium (CrIII), generating intermediate chromium compounds and reactive oxygen species (ROS). High concentrations of ROS result in oxidative stress, which has negative effects on lipids, enzymes, and DNA, thereby altering cell function and stimulating apoptosis [19].

Studies have shown that CrVI mainly induces apoptosis through the mitochondrial pathway [2,20]. This process involves the *Bax* (B-cell lymphoma protein 2- associated X) and *Bcl2* (B-cell lymphoma protein 2) proteins, which regulate mitochondrial membrane permeability [21]. *Bcl2* is an anti-apoptotic protein that blocks apoptosis, whereas *Bax* is a pro-apoptotic protein that stimulates it by interacting with proteins in the mitochondrial membrane. This modifies the organelle’s permeability and ultimately releases the enzymes responsible for apoptosis [22]. In vitro and in vivo studies provide evidence that CrVI induces oxidative stress associated with lipid peroxidation, activation of protein kinase C, DNA fragmentation, altered gene expression and apoptotic cell death [1,23].

Lately, interest in natural products, particularly as alternatives to pharmaceuticals, has increased significantly. Consequently, research has become more focused on studying the effects of bioactive plant compounds [24].

*Hypericum perforatum*, commonly known as St. John’s wort, is a widely used plant valued for the beneficial effects of its active compounds: naphthodianthrones (e.g., hypericin and pseudohypericin), phloroglucinols (e.g., hyperforin and adhyperforin), biflavonoids (e.g., biapigenin and amentoflavone), glycosides (e.g., rutin and quercetin) and other flavonoids [25]. The *Hypericaceae* family comprises over 1000 species [26], but extracts from *Hypericum perforatum* exhibit the most potent antioxidant activity. The antioxidant activity of *Hypericum perforatum* has been demonstrated in several in vivo experimental models in rats and mice, where administration of the plant extract reduced oxidative stress markers and enhanced endogenous antioxidant defences in the brain, liver, and peripheral nerves [27,28,29]. The underlying antioxidant mechanism of *Hypericum perforatum* has been attributed to multiple actions, including free-radical scavenging, metal-chelating capacity, and quenching of reactive oxygen species [30]. In this context, flavonoids and/or caffeoylquinic acids abundantly present in *Hypericum perforatum* extracts are considered the major contributors to free-radical scavenging and the reduction of lipid peroxidation [31,32].

Consistently, standardised *Hypericum perforatum* preparations showed relevant antioxidant effects both in vitro and in vivo by inhibiting free-radical generation and lipid peroxidation in PC12 cells [33]. In addition, extracts of *Hypericum perforatum* have been shown to counteract cytokine-induced shifts in pro- and anti-apoptotic regulators by suppressing the transcription of pro-apoptotic genes while maintaining the activity of anti-apoptotic Bcl-2 family members. Through this mechanism, they interfere with multiple phosphorylation events within cytokine signalling cascades, ultimately limiting the expression of inflammatory and apoptotic genes [34]. Extracts of *Hypericum perforatum* were observed to protect pancreatic β-cells by preventing apoptosis in cultured rat and human islet cell lines [35,36]. Furthermore, the extract was shown to attenuate the progression of acute pancreatitis [37].

The effects of CrVI contamination primarily impact the health of plants, aquatic and terrestrial animals, and then humans, as heavy metals enter the body through both direct exposure and the food chain [19]. Once inside the body, hexavalent chromium accumulates in tissues such as bones, spleen, heart, kidneys, liver, the gastrointestinal system [38], the pituitary gland [7], lungs and brain [39], and male reproductive organs [40]. As environmental contamination continues to rise—driven by the rapid expansion of industries that use hexavalent chromium—and given this metal’s capacity to function as an endocrine disruptor, we sought to build upon our research team’s previous work by closely examining its structural and functional impact on the endocrine pancreas. Furthermore, we assessed whether the bioactive compounds in St. John’s wort can mitigate the oxidative effects associated with CrVI exposure.

## 2. Results

### 2.1. HPLC Analysis

**Figure 1:**
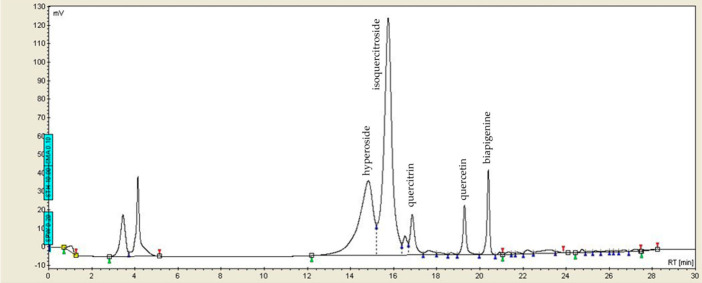
The HPLC chromatogram of the reference standard St. John’s wort dry extract HRS3 at 360 nm.

The analysis of the standard St. John’s wort dry extract (HS3) was carried out using liquid chromatography (HPLC) and the resulting chromatogram is shown in Figure 1. Comparing the obtained chromatogram with the manufacturer’s provided chromatogram revealed the presence of hyperoside (tR = 14.825 min), isoquercitroside (tR = 15.748 min), quercitrin (tR = 16.865 min), quercetin (tR = 19.28 min), and biapigenine (tR = 20.395 min).

**Figure 2:**
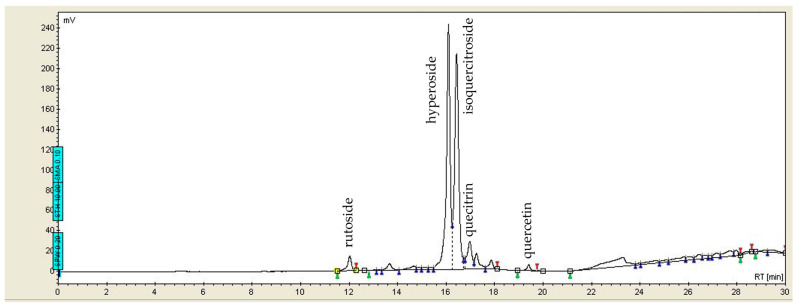
The HPLC chromatogram of the *Hypericum perforatum* extract at 360 nm.

In the chromatogram of the aqueous *Hypericum perforatum* extract (Figure 2), only two major peaks are observed at retention times of 16.098 and 16.433 min. A comparison with the standard extract indicates that these substances may be attributed to hyperoside and isoquercitroside, highly water-soluble flavonoids that exhibit excellent water-extractability. Peaks of smaller size have also been identified at retention times of 12.027, 16.977, and 19.405 min, and are attributed to rutoside, quercitrin, and quercetin, respectively.

### 2.2. Histological Assessment

**Figure 3:**
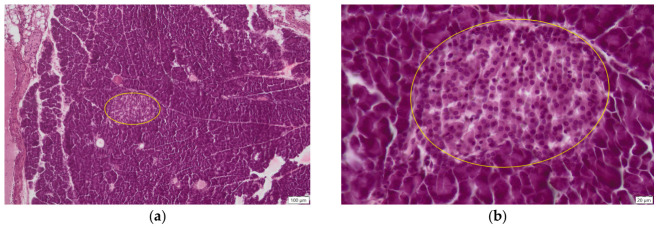
Pancreas—Control group, histological sections, H.E. staining, (**a**) normal aspect of pancreas: endocrine part (Langerhans islet, yellow circle) surrounded by exocrine part (acini), ob. 10×; (**b**) normal Langerhans islet (yellow circle) ob. 40×.

The microscopic examination of the pancreas in the control group (C) revealed a normal histological structure (Figure 3), consisting of an exocrine and an endocrine component. The exocrine portion, responsible for pancreatic juice secretion, represented the predominant part of the gland and was composed of pyramidal-shaped acinar cells. These cells were of serous type, exhibiting a centrally located spherical nucleus and an intensely stained cytoplasm. The apical pole of the acinar cells delineated a lumen through which the secretory product was discharged. The cells were arranged into spherical units called acini that surrounded the endocrine structures of the pancreas.

The endocrine pancreas was represented by the islets of Langerhans, which displayed a normal morphology, appearing as oval structures composed of endocrine cells. Four cell types are identified within the islets, two of which are directly involved in glucose homeostasis: A (alpha) cells, which synthesise glucagon, and B (beta) cells, which secrete insulin. Insulin is a peptide hormone with a hypoglycemic effect, whereas glucagon, its physiological antagonist, exerts a hyperglycemic effect.

**Figure 4:**
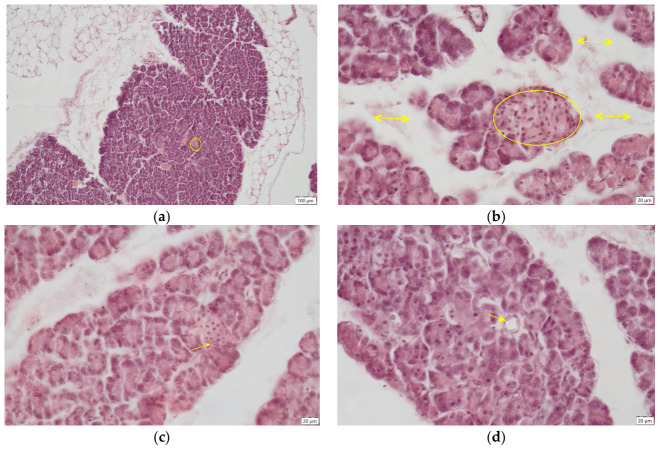
Pancreas, Cr group—histological sections H.E. staining, (**a**) small endocrine islet (circle), ob. 10×; (**b**) interstitial oedema (arrows), small islet (circle), ob. 40×; (**c**) degenerative lesions of the Langerhans islets: cytoplasm vacuolisation, pycnotic nuclei (arrow), ob. 40×; (**d**) swelling of endothelial cells (arrow) ob. 40×.

**Figure 5:**
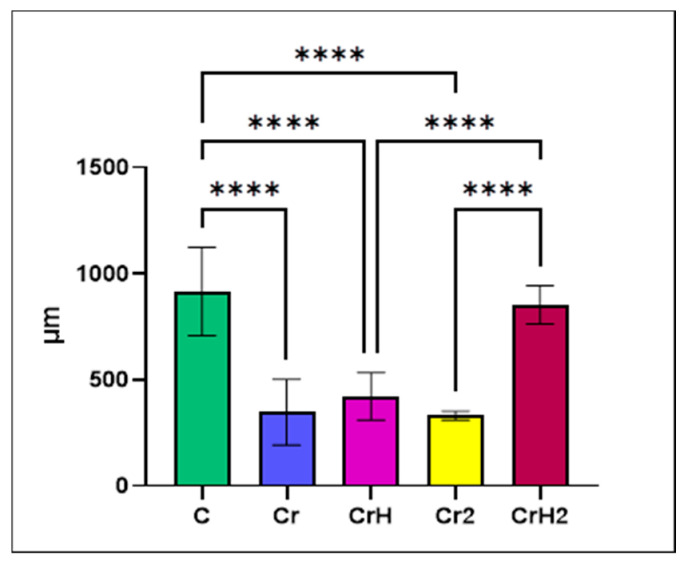
The perimeter of the islets of Langerhans (µm; **** *p* < 0.0001).

In the group exposed to hexavalent chromium for three months (Cr group), interlobular and severe intralobular oedema was observed, absent in the control group, accentuating the delimitation of pancreatic lobules, occurring around exocrine acini, or even isolating the islets of Langerhans from the surrounding exocrine tissue (Figure 3a). Additionally, the size of the islets of Langerhans was significantly (*p* < 0.0001) reduced relative to the control group (Figure 4a,b and Figure 5). Areas of islet atrophy were evident, in some regions leaving only residual spaces where endocrine structures had previously been present. Degenerative changes in endocrine cells were also noted, including cytoplasmic vacuolisation and pycnotic nuclei (Figure 4c). Chromium exposure-induced alterations in endothelial cells, manifested by cellular hypertrophy and swelling (Figure 4d).

**Figure 6:**
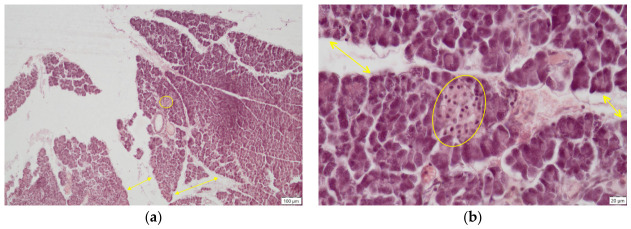
Pancreas, CrH group—histological sections, H.E. staining, (**a**) small Langerhans islet (circle), oedema, ob. 10×; (**b**) Langerhans islet (circle) with degenerative lesions, interstitial oedema (arrows), ob. 40×.

In animals receiving hexavalent chromium concomitantly with aqueous extract of *Hypericum perforatum* (CrH group), pancreatic lesions persisted, with interlobular oedema still present. Following consecutive administration of chromium and *Hypericum perforatum*, alterations in the size of the islets of Langerhans were observed. Although islet dimensions remained significantly (*p* < 0.0001) smaller than those in the control group, a modest, non-significant increase was noted compared with the groups (Cr and Cr2) exposed to chromium alone (Figure 4, Figure 5 and Figure 6).

**Figure 7:**
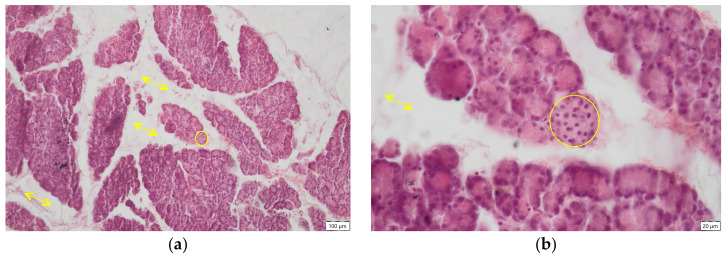
Pancreas, Cr2 group—histological sections, H.E. staining, persisting interstitial oedema (arrows), very small Langerhans islet (circles), (**a**) ob. 10× and (**b**) 40×.

Cessation of chromium administration and continuation with distilled water alone (Cr2 group) for an additional month did not improve the pancreatic architecture. Oedema persisted, with both interlobular and pronounced intralobular oedema appearing even more severe than in the Cr-treated group. One month after cessation of chromium exposure, the islets of Langerhans remained significantly (*p* < 0.0001) smaller than those in the control group. Although administration of the toxicant was stopped, no recovery in islet size was observed; instead, islet dimensions were slightly, but not significantly, reduced compared with those in the Cr and CrH group (Figure 7).

**Figure 8:**
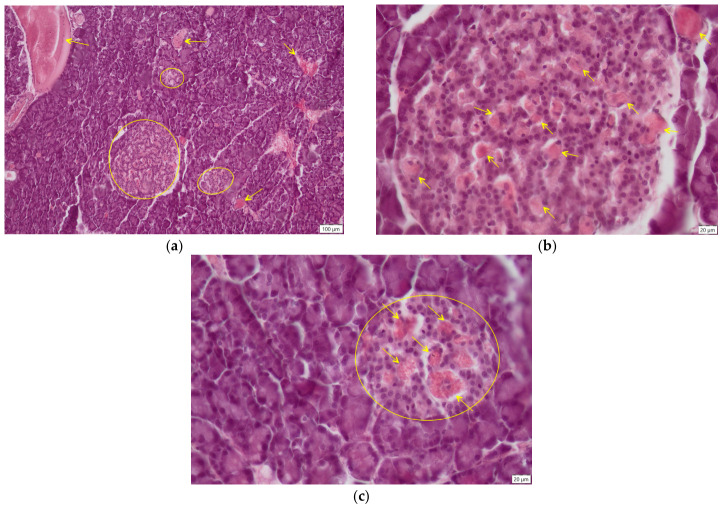
Pancreas, CrH2 group—histological sections, H.E. staining, (**a**) Langerhans islets (circles), dilation of blood vessels (arrows), ob. 10×; (**b**) large Langerhans islet with a lot of dilated blood vessels (arrows), ob. 40×; (**c**) Langerhans islet (circle) with pronounced vasodilation (arrows), ob. 40×.

However, substitution of hexavalent chromium after three months of exposure with the aqueous extract of *Hypericum perforatum* (CrH2 group) resulted in significant structural improvement of the pancreas. The overall appearance of the organ was compact, closely resembling that of the control group (Figure 8a). No oedema was observed between lobules or within the exocrine and endocrine components. Administration of *Hypericum perforatum* for one month after cessation of chromium exposure resulted in marked changes to the histoarchitecture of the endocrine pancreas. Notably, the size of the islets of Langerhans was significantly (*p* < 0.0001) increased compared with all chromium-exposed groups (Cr, CrH, and Cr2), with islet dimensions approaching those observed in the control group, suggesting a structural recovery (Figure 8a). A striking feature was the presence of numerous dilated blood vessels, engorged with blood (Figure 8b). The marked vasodilatation was particularly evident within the islets of Langerhans (Figure 8c).

As shown in Table 1, oedema was minimal in control animals and increased markedly in the CrVI group. In contrast, oedema severity was reduced in the *Hypericum perforatum*-treated groups. In the simultaneous administration group, the oedema score decreased to 1, and in the post-exposure treatment group, it was completely absent (score 0), demonstrating a clear ameliorating effect of the plant extract.

### 2.3. Gene Expression Analysis

**Figure 9:**
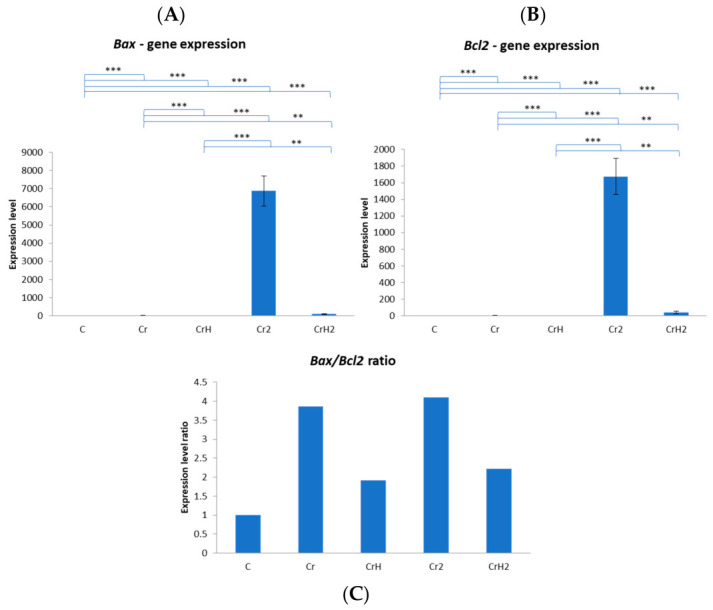
Relative mRNA expression of apoptosis-related genes in pancreatic tissue. Relative expression levels are presented as fold change versus the control group, calculated using the 2^−ΔΔCt^ method with *GAPDH* as the reference gene. Data are presented as mean ± SD (*n* = 6 per group). Statistical analysis was performed using one-way ANOVA followed by Tukey’s post hoc test. ** *p* < 0.01, *** *p* < 0.001 versus control or as indicated.

Quantitative PCR analysis revealed significant alterations in apoptosis-related gene expression among the experimental groups (Figure 9). For *Bax*, the pro-apoptotic marker (Figure 9A), expression levels were very low in the control (C) group and remained at similarly low levels in the Cr, CrH, and CrH2 groups. By contrast, animals from the Cr2 group (chromium exposure followed by withdrawal) exhibited a pronounced overexpression of *Bax*, which was significantly higher than in all other groups (*p* < 0.001) as confirmed by Tukey’s post hoc test following one-way ANOVA (*p* < 0.001). The high fold-change values observed in the Cr2 group reflect the normalisation to low baseline expression levels in the control group and represent relative (not absolute) transcript abundance. In the case of *Bcl2*, the anti-apoptotic gene (Figure 9B), a comparable pattern was observed. Expression remained weak in the control, Cr, and CrH groups, while Cr2 animals displayed a strong overexpression of *Bcl2* transcripts (*p* < 0.001 vs. all other groups). The CrH2 group showed a modest increase in *Bcl2* expression compared to controls, although this remained clearly lower than the levels recorded in Cr2.

The *Bax*/*Bcl2* ratio (Figure 9C) provided a more integrative assessment of the apoptotic balance. In the control group, the ratio was close to equilibrium, reflecting the physiological balance between pro- and anti-apoptotic signals. Exposure to chromium alone (Cr) shifted this ratio upwards, indicating that even in the absence of marked changes in absolute gene expression, the apoptotic pathway tended toward activation. The imbalance became most evident in the Cr2 group, where the ratio reached its highest levels, indicating that chromium withdrawal without therapeutic intervention did not restore homeostasis but instead reinforced the pro-apoptotic drive. In contrast, both preventive (CrH) and therapeutic (CrH2) administration of *Hypericum perforatum* extract reduced the *Bax*/*Bcl2* ratio compared to chromium exposure alone. In the CrH2 group, the ratio approached control values, suggesting a clear protective effect of the plant extract in re-establishing the apoptotic balance.

### 2.4. Blood Glucose Levels

**Figure 10:**
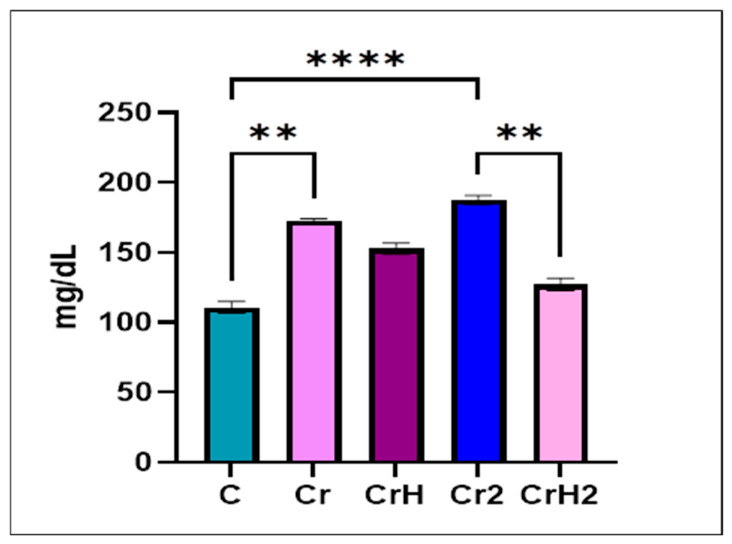
Effects of CrVI exposure and *Hypericum perforatum* treatment on blood glucose levels. Blood glucose concentrations (mg/dL) were measured at the end of the experimental period. Data are presented as mean ± SD (*n* = 6 per group). Statistical analysis was performed using one-way ANOVA followed by Tukey’s post hoc test. ** *p* < 0.01, **** *p* < 0.0001 versus control or between indicated groups.

Blood glucose levels increased in all experimental groups compared to the control group (Figure 10), but the highest significant increase was recorded in the groups exposed only to CrVI (Cr group—*p* < 0.01, and Cr2 group—*p* < 0.0001). In the groups treated with *Hypericum perforatum* extract as a preventive measure (CrH group), a decrease in glucose levels was observed compared to the Cr group exposed only to chromium. The largest decrease was observed in the CrH2 therapeutic group, which was significantly lower (*p* < 0.01) than the Cr2 group. In this case, blood glucose values are close to normal.

Subchronic exposure to hexavalent chromium (Cr group) induced marked pancreatic damage, characterised by interlobular and severe intralobular oedema, endocrine cell degeneration, islet atrophy, and a significant reduction in islet area. Although *Bax* and *Bcl2* expression levels remained low, the *Bax/Bcl2* ratio shifted toward a pro-apoptotic profile. These changes were accompanied by a significant increase in blood glucose levels, reflecting endocrine dysfunction.

In the Cr2 group, chromium withdrawal without therapeutic intervention failed to restore pancreatic homeostasis. Structural damage persisted, with the smallest islet area among all groups. This group exhibited the strongest pro-apoptotic imbalance, marked by pronounced *Bax* overexpression and the highest *Bax/Bcl2* ratio, along with the greatest increase in blood glucose levels, indicating exacerbated apoptotic and metabolic disturbances.

Preventive administration of *Hypericum perforatum* extract during chromium exposure (CrH group) partially attenuated chromium-induced alterations. Although histological lesions persisted, the islet perimeter increased compared to the Cr and Cr2 groups. The *Bax/Bcl2* ratio was reduced, and blood glucose levels decreased relative to chromium-only exposure, suggesting an initial protective effect on pancreatic endocrine function.

The most pronounced recovery was observed in the CrH2 group, where *Hypericum perforatum* treatment followed chromium exposure. This group showed substantial structural improvement, absence of oedema, enlarged islets, and enhanced vascularisation. *Bax* levels remained low, *Bcl2* expression modestly increased, and the *Bax/Bcl2* ratio approached control values. Functionally, blood glucose levels were markedly reduced and close to normal.

Overall, chromium exposure induced severe pancreatic injury that was not reversed by exposure withdrawal alone, whereas *Hypericum perforatum* exerted both preventive and especially therapeutic effects, contributing to restoration of apoptotic balance, pancreatic structure, and glucose homeostasis.

## 3. Discussion

Heavy metals are non-biodegradable and can bioaccumulate in soil, water, and air, posing a serious threat to human health. Chromium exists in several oxidation states, but hexavalent chromium is the most hazardous due to its high solubility and oxidative potential [41], as well as its ability to easily cross the plasma membrane via phosphate and anion transporters [42]. Once in the body, CrVI undergoes sequential reduction reactions as part of a detoxification mechanism. During intracellular CrVI reduction, reactive oxygen species are generated, contributing to oxidative toxicity [43]. Although ROS can damage cellular structures, antioxidant systems act to neutralise them and protect cells against oxidative stress [44]. Thus, antioxidants play a crucial role in moderating CrVI-induced cytotoxicity and are essential to the detoxification process [45,46]. When ROS production exceeds antioxidant capacity, oxidative stress occurs. Both in vitro and in vivo studies confirm that CrVI induces oxidative stress [1], leading to lipid peroxidation, protein kinase C activation, DNA fragmentation, altered gene expression, and apoptosis [23].

In our study, three months of CrVI exposure revealed modifications in pancreas histoarchitecture. Structural alterations in the pancreas mainly arise from the accumulation of this heavy metal in the organ, as shown by Solis-Heredia et al. [16]. These modifications included interlobular and severe intralobular oedema that enhanced delineation of pancreatic lobules or appeared around exocrine acini, in some cases even separating the islets of Langerhans from the adjacent exocrine tissue. Furthermore, additional alterations were characterised by a significant reduction in the size of the islets of Langerhans (*p* < 0.0001), atrophy of the endocrine islets, and degenerative changes in endocrine cells. Similar lesions were documented by Srikanta Guria et al. [18] subsequent to intraperitoneal administration of CrVI at a dosage of 24 mg/kg body weight over a duration of 21 days in mice. Electron microscopy of the endocrine pancreas in rats exposed subcutaneously to 50 mg/kg b.w. of CrVI revealed nuclear membrane fragmentation, mitochondrial vacuolisation, marked enlargement of the Golgi complex and endoplasmic reticulum, as well as a reduced number of secretory granules [17]. The aforementioned structural modifications in endocrine cells signify a compromised ability for the synthesis and secretion of insulin, which are indicative of pathologies observed in diabetic organisms [47,48]. Conversely, García-Niño et al. [49] did not observe any histological changes to the structure of the pancreas, which is probably due to the low dose of chromium, 15 mg/kg b.w. single intraperitoneal injection. Following exposure to chromium, endothelial cell swelling was also observed. As demonstrated by Zhang et al. [22], exposure of human Hep3B hepatoma cell line to 20 µM CrVI for 12 h has been found to inhibit Na+K+ATPase, resulting in alterations to cell volume and the ability of water to enter and be retained within the cell, leading to swelling of endothelial cells [50]. However, CrVI-induced disruption of the Na^+^/K^+^ pump activity will also affect Ca^2+^ homeostasis [9], promoting their influx and therefore causing cell swelling [51].

Several studies have indicated that CrVI toxicity induces apoptosis primarily via intrinsic mitochondrial mechanisms in various human tumour cell lines [20], nephrocytes [2] and human prostate cells [52,53]. Similar findings have been reported in rat models, including the anterior pituitary [7], liver [54], kidney [55], placenta [56], ovaries [57], uterus [58], thyroid gland [9], and adrenal gland [15]. Mitochondria are target organelles for CrVI [19], the permeability of whose membrane is controlled by *Bax* and *Bcl2* proteins [21].

In our study, exposure to hexavalent chromium (Cr group) reduced the expression levels of both *Bax* and *Bcl2*. However, the *Bax/Bcl2* ratio increased, indicating that the apoptotic pathway was inclined towards activation despite minimal changes in absolute gene expression. These data do not coincide with those from studies conducted on cell cultures and kidneys, which describe changes in *Bax* and *Bcl2* protein expression [2,20,22,55], probably due to the experimental model, the doses used and the targeted organ. One month after chromium withdrawal (Cr2 group), microscopic lesions persisted, with some appearing even more pronounced. Consequently, the interlobular and pronounced intralobular oedema persisted, and the size of the islets was slightly, but not significantly, reduced compared with the Cr group. These observations were supported by molecular data, showing marked overexpression of both *Bax* and *Bcl2* genes, significantly higher than in all other groups (*p* < 0.001). The combination of increased *Bax* expression and reduced *Bcl2* protein levels indicates activation of the intrinsic mitochondrial apoptotic pathway in the pancreas of the Cr2 group. The *Bax/Bcl2* ratio reached its highest values, suggesting that chromium withdrawal without therapeutic intervention failed to restore homeostasis and instead enhanced the pro-apoptotic drive.

Antioxidants represent an essential category of biomolecules that protect cellular components from oxidative damage induced by heavy metals [19]. They function by inhibiting the oxidation of biomolecules through the stimulation of protective enzymes and proteins and by scavenging superoxide radicals, thereby establishing themselves as pivotal agents in the detoxification of ROS [59]. The bioactive profile of *Hypericum perforatum* comprises multiple classes of phytochemicals; however, within the aqueous extract, only specific compound groups are consistently detected [26]. A major group in aqueous preparations is the flavonoid glycosides, whose high water solubility makes them key contributors to the extract’s biological activity. This category includes hyperoside (quercetin-3-O-galactoside), isoquercitrin (quercetin-3-O-glucoside), rutin (quercetin-3-O-rutinoside), quercitrin (quercetin-3-O-rhamnoside), and quercetin. These flavonoids, detected in the St. John’s wort extract used, are widely recognised in the literature as the main water-soluble antioxidants of the plant [60,61]. In contrast, naphthodianthrones—mainly hypericin and pseudohypericin—are present only in minimal concentrations, and were not detected in our extract. Their low solubility in water and preferential extraction into organic solvents result in very limited recovery [62]. Furthermore, phloroglucinols such as hyperforin and adhyperforin are almost entirely absent from aqueous preparations, as shown in the chromatogram (Figure 2). Hyperforin, in particular, exhibits high instability in water and is therefore practically not extracted into aqueous extracts [26,60].

Hyperoside, a major component identified in the aqueous extract, exhibits antioxidant properties primarily by scavenging ROS [63]. It also shows notable DPPH radical-quenching activity, inhibits lipid peroxidation [64], and suppresses nitric oxide synthase in a concentration-dependent manner, as observed in rat cerebral homogenate and blood [65]. In addition, hyperoside has been reported to prevent apoptosis by improving mitochondrial function, lowering mitochondrial membrane potential disruption, decreasing ROS production, and limiting the release of cytochrome C from mitochondria [66].

Rutin, another constituent, provides protection against oxidative stress through its DPPH radical-scavenging activity [64,67,68]. Its antioxidant capacity has been shown to be strong in vitro, demonstrated by inhibition of lipid peroxidation and effective scavenging of superoxide radicals and hydrogen peroxide [68]. Song et al. [69] also reported that rutin can decrease *Bax* protein expression while increasing *Bcl-2* levels in HT22 cells. In HepG2 cells, it has been shown to enhance GSH activity [70]. Moreover, in vivo studies in Wistar rats confirmed the antioxidant action of rutin, as reflected in the stimulation of GSH, GPx, GR, SOD, and CAT activities [71].

Although quercetin is present in relatively small amounts in the aqueous extract of *Hypericum perforatum*, its antioxidant effect is significant. It provides protection against hydrogen peroxide-induced damage [72]. In human SH-SY5Y cells, quercetin was shown to suppress apoptosis by reducing DNA fragmentation, inhibiting the caspase cascade and the expression of the pro-apoptotic protein *Bax*, while increasing the expression of the anti-apoptotic protein *Bcl-2* [73]. Oxidative stress–related effects, such as mitochondrial lipid peroxidation and loss of mitochondrial transmembrane potential, were also attenuated in the presence of quercetin [74]. Its antioxidant activity is likewise outstanding in vitro, with quercetin considered one of the most potent ROS scavengers among flavonoids [75]. In addition, quercetin exhibits strong DPPH radical-scavenging activity [64,67,76], and shows an inhibitory effect on nitric oxide synthase [65].

The antioxidant activity of quercitrin surpasses that of quercetin, likely due to its greater bioavailability in the digestive tract [77]. Its effects are mediated through mechanisms similar to those of the other constituents, including DPPH radical-scavenging activity and inhibition of lipid peroxidation [64,78].

In our study, after three months of concomitant exposure to CrVI and *Hypericum perforatum* aqueous extract (CrH group), interlobular oedema remained present, and the islets of Langerhans were still significantly smaller (*p* < 0.0001) than those in the control group; however, a modest, non-significant increase was observed compared with the chromium-only groups (Cr and Cr2). When CrVI was replaced with the aqueous extract for one month (CrH2 group), pancreatic architecture improved, showing a compact appearance without oedema, similar to the control group. Consistent with these findings, other studies have also documented the anti-inflammatory effects of St. John’s wort in diabetes, mediated either through modulation of prostaglandin E2 formation [79,80], or through inhibition of pro-inflammatory cytokines and related factors [81,82]. Islet size was significantly increased (*p* < 0.0001) compared with all chromium-exposed groups (Cr, CrH, and Cr2), with values approaching those of the control group, indicating structural recovery. However, many dilated, blood-filled vessels were also observed.

When tissue damage occurs, the body’s initial response is vasodilation. This increases blood flow, ensuring the supply of immune cells and nutrients, which are necessary for the removal of dead cells and toxins, and for the initiation of the healing process [83]. Bioactive plant compounds, such as flavonoids and phenolic acids, can induce vasodilation through various mechanisms. A primary mechanism involves the generation of endothelium-derived vasodilatory mediators. Oxidative stress reduces the bioavailability of these mediators. Therefore, the antioxidants in *Hypericum perforatum* help maintain the vasodilatory effect by neutralising free radicals [84].

The *Bax* protein evolved similarly in the *Hypericum perforatum-*treated groups (CrH and CrH2), remaining at a low level. Expression of *Bcl2* remained low when *Hypericum perforatum* was administered together with CrVI. However, in the CrH2 group, expression increased slightly, although it remained below the level observed in the Cr2 group, which was exposed only to CrVI and water. The *Bax/Bcl2* ratio was reduced by the administration of *Hypericum perforatum* extract, both in a preventive (CrH) and a therapeutic (CrH2) way, compared to chromium exposure alone. The ratio in the CrH2 group was close to the control values, indicating that the plant extract had a clear protective effect in restoring the apoptotic balance.

Our primary objective was to evaluate the extract’s protective and therapeutic effects against chromium-induced toxicity, rather than to assess its independent impact on metabolism. A limitation of this study is the absence of a group treated solely with *Hypericum perforatum* extract. However, previous studies have reported that administering comparable doses of *H. perforatum* extract to healthy rodents does not alter blood glucose levels or induce cellular changes associated with apoptosis [34,35,36]. Because of this, a control group receiving only distilled water was an appropriate baseline. The CrH and CrH2 groups were then used to examine how the extract worked both as a preventive measure and as a treatment after exposure.

Reactive oxygen species, including hydrogen peroxide and hydroxyl radicals, are capable of damaging biomolecules and eventually triggering apoptosis [85]. Consequently, eliminating excess ROS or limiting their generation through antioxidant activity may help prevent cell death. Benedí et al. [33] reported that hydrogen peroxide decreased the viability of PC12 cell cultures, while the presence of *Hypericum perforatum* extract mitigated its cytotoxic effects. A comparable anti-apoptotic and protective effect of St. John’s wort has also been reported in human SK-N-MC cells [86]. This effect is likely due to the fact that *Hypericum perforatum*, via its phytoconstituents, operates both through direct free-radical scavenging [87] and by modulating enzymes associated with superoxide (O_2_^−^) radical neutralisation. Furthermore, St. John’s wort can quench nitrogen-centred radicals such as DPPH, as well as oxygen-centred radicals, including hydroxyl radicals. The capacity of its bioactive compounds to reduce and scavenge DPPH indicates their role as electron donors that interact with free radicals, converting them into more stable species and thereby interrupting radical chain reactions [33]. In addition, *Hypericum perforatum* may chelate divalent metal ions through the hydrogen-donating properties of hydroxyl groups within its molecules [87].

The impaired endocrine function of the pancreas in this study is further supported by elevated blood glucose levels, which were significantly higher in the chromium-exposed groups (Cr and Cr2) than in all other groups (C, CrH, and CrH2). Hyperglycemic responses after hexavalent chromium exposure have likewise been reported in experimental studies involving mice [16,41], rats [88,89], and rabbits [90]. The hyperglycemia detected in rats exposed to CrVI may be attributed to reduced insulin levels resulting from the structural lesions described, as well as to chromium-induced mitochondrial dysfunction [20]. The literature highlights inflammation and mitochondrial dysfunction as key contributors to endocrine cell apoptosis [91,92]. Mitochondrial impairment, in turn, contributes to the development of diabetes by disrupting energy metabolism, promoting insulin resistance, and ultimately compromising endocrine cell function [93]. Energy homeostasis, achieved by promoting cellular glucose uptake and mitochondrial biogenesis, is governed by AMP-activated protein kinases (AMPKs) [94]. Upon activation, AMPK plays a key role in the regulation of blood glucose levels [95]. Reduced glucose bioavailability stimulates enzyme activation. Activated AMPK inhibits gluconeogenesis, thereby decreasing hepatic glucose output and ultimately lowering blood glucose concentrations [96]. In vitro studies suggest that antioxidants, such as those present in *Hypericum perforatum*, may activate these enzymes [94,97,98,99]. In the present study, a reduction in blood glucose was observed exclusively following treatment with the St. John’s wort extract, with values in the CrH2 group approaching normal physiological levels. The hypoglycemic, even antidiabetic, effects of *Hypericum perforatum* have been demonstrated in several studies conducted in diabetic animal models [82,100,101,102,103,104]. Bioactive compounds from St. John’s wort have been shown to enhance insulin secretion [100], reduce insulin resistance [105], and modulate the activity of enzymes involved in gluconeogenesis and carbohydrate metabolism [101,106].

Evidence from previous studies suggests that *Hypericum perforatum*, through its active constituents and also by interfering with the gluconeogenesis pathway, can modulate carbohydrate metabolism [106]. Quercetin, for example, has been shown to reduce glucose-6-phosphatase activity [107], thereby decreasing glucose production in hepatocytes [99]. Another component, rutin, lowers hepatic and renal G6Pase activity in diabetic rats [108]. Hyperoside has likewise been reported to reduce the activity of key gluconeogenic enzymes in diabetic rats [109]. Phenolic compounds with antidiabetic effects, including flavonoids, typically contain several hydroxyl groups, a double bond, and a C-4 ketonic functional group [110]. Flavonoids possessing a greater number of hydroxyl groups, such as quercetin, exhibit superior antidiabetic activity, likely due to their enhanced capacity to form hydrogen bonds with target molecules [111]. Taken together, these findings support the hypothesis that the glucose-lowering effect observed in this study may also be attributed to the capacity of *Hypericum perforatum* bioactive compounds to modulate the gluconeogenic pathway.

Protection of pancreatic endocrine cells through reduction in ROS levels and preservation of their functional capacity, including insulin synthesis, may be mediated by antioxidants such as flavonoids. Preclinical studies have shown that these compounds can alleviate endocrine cell dysfunction and enhance insulin secretion [112]. Consistent with this mechanism, the ability of St. John’s wort bioactive constituents to suppress ROS generation has been demonstrated in both in vivo and in vitro models [113]. Studies show that St. John’s wort extract protects the endocrine islets of the pancreas against cytokine-induced apoptosis [35], which are responsible for stimulating the expression of pro-apoptotic genes, leading to apoptosis [36]. Furthermore, studies conducted on isolated endocrine cell cultures or beta cell cultures have demonstrated that *Hypericum perforatum* has the ability to inhibit apoptosis and prevent insulin secretion deficiency [34,35,36]. The prophylactic use of St. John’s wort has been shown to ameliorate diabetes in INS-1 cell cultures (rat insulinoma cell line). Reported effects include reduced loss of endocrine cells, preservation of islet mass, improved insulin secretion, enhanced pancreatic antioxidant capacity, and prevention of endocrine cell apoptosis [114]. Research has shown that the bioactive compounds in St. John’s wort also possess insulinotropic properties [114], potentially through inhibition of pancreatic poly(ADP-ribose) polymerase. In diabetic male Wistar rats, treatment with St. John’s wort extract has been associated with this mechanism, involving reduced apoptosis of endocrine cells and enhanced insulin synthesis and secretion, leading to improved carbohydrate metabolism, increased insulinotropic activity, and better regulation of glucose production [106].

The two intervention strategies demonstrated different levels of efficacy in mitigating chromium-induced pancreatic injury. Simultaneous administration of *Hypericum perforatum* during chromium exposure (CrH group) produced a partial protective effect. Although histological lesions such as oedema and endocrine degeneration were still present, improvements were observed compared with the chromium-only groups, including an increased islet perimeter, a reduced *Bax/Bcl2* ratio, and lower blood glucose levels. These findings suggest that preventive administration may attenuate the early stages of chromium-induced pancreatic damage and help maintain partial endocrine function.

In contrast, post-exposure administration of *Hypericum perforatum* (CrH2 group) resulted in the most pronounced recovery. This intervention was associated with substantial structural restoration of pancreatic tissue, including the absence of oedema, enlarged islets, and improved vascularisation. At the molecular level, apoptotic balance was markedly improved, as indicated by low *Bax* expression, a modest increase in *Bcl2* levels, and a *Bax/Bcl2* ratio approaching control values. Functionally, blood glucose levels were significantly reduced and nearly normalised. These results indicate that therapeutic administration after toxic exposure may be more effective in promoting tissue recovery and restoring pancreatic endocrine function.

These findings suggest that while simultaneous administration may have preventive value in individuals at risk of chromium exposure, post-exposure treatment may be more therapeutically relevant in clinical situations where exposure has already occurred and tissue injury is established. Therefore, *Hypericum perforatum* may have potential as both a protective agent and a therapeutic intervention, with greater efficacy when administered after the toxic insult.

Although the present study demonstrates that *Hypericum perforatum* extract attenuates CrVI-induced oxidative damage in the endocrine pancreas in an animal model, direct extrapolation of these findings to humans remains uncertain. Species-related differences in physiology, metabolism, immune responses, and genetic background may influence both toxicity and protection, limiting the prediction of efficacy and safety in humans, particularly under chronic exposure conditions or in individuals with metabolic comorbidities. Moreover, the experimental design does not fully reflect real-life human exposure, which typically involves multiple exposure routes and long-term, low-dose contact. Acute or subchronic exposure models in animals may exaggerate oxidative stress responses, potentially overestimating both toxic and protective effects and thereby limiting the ecological validity of the findings.

Future studies should evaluate the antioxidant efficacy of isolated bioactive constituents of *Hypericum perforatum* relative to the crude extract to determine whether the observed protective effects against CrVI are attributable to specific compounds or to synergistic interactions among multiple constituents. Furthermore, validation of these findings in human pancreatic β-cell lines or, where available, in human pancreatic islets is warranted to improve the translational relevance of the results.

## 4. Materials and Methods

The experimental protocol was reviewed and approved by the Ethical Committee of the Banat University of Agricultural Sciences and Veterinary Medicine “King Michael I of Romania,” Timișoara (Approval No. 60/23.07.2019).

The animals used in this study were obtained from the University of Medicine and Pharmacy “Victor Babeş,” Timișoara, Romania, an authorised animal supplier. Upon arrival, 30 healthy adult male Wistar rats (220–240 g) were housed under laboratory conditions (temperature: 22 ± 2 °C; relative humidity: 40–60%; light/dark cycle: 12 h/12 h) and allowed a one-week acclimatisation period. Throughout the experiment, the rats were provided with a standard Biovetimix diet (Biovet, Tanacu, Romania, code 140-501) and had unrestricted access to food and water. Animals were housed in standard polycarbonate cages (dimensions: 750 × 720 × 360 mm).

Potassium dichromate (K_2_Cr_2_O_7_), recognised as the most soluble salt of chromium, was procured from Sigma-Aldrich (Darmstadt, Germany) and employed as the source of hexavalent chromium in the present study. The compound was of analytical grade (≥99% purity) and stored in tightly sealed containers under dry conditions at room temperature, protected from direct light and moisture, to preserve stability. Potassium dichromate was diluted to 75 mg/L in distilled water.

The EPA has established an LOAEL of 25 mg/L for CrVI. The stock solution ranged from 1000 mg/L to 10% (*w*/*v*). Based on existing data regarding chromium toxicity, a working solution of 75 mg/L of K_2_Cr_2_O_7_ was selected for this experimental protocol to ensure reproducibility and to enhance the manifestation of chromium-related toxic effects within the experimental model [4].

Dried flowers of *Hypericum perforatum* were procured from a certified natural products supplier (Dacia Plant, Romania) and used as the raw material for aqueous extract preparation. The extraction procedure was adapted from previously reported methods [115].

Briefly, the dried flowers were accurately weighed and mixed with distilled water at a 0.25/10 (*w*/*v*) ratio. The mixture was heated to 90 °C and maintained at this temperature for 10 min under constant stirring to facilitate the release of water-soluble bioactive compounds. Following thermal treatment, the suspension was allowed to cool to room temperature and subsequently filtered through standard laboratory-grade filter paper to remove plant residues. The extract was stored in sterile amber glass containers at 4 °C in order to minimise degradation of thermolabile polyphenolic constituents.

The experimental protocol (Table 2) was conducted on five groups of rats, each consisting of six animals (*n* = 6), which were assigned to the following treatment regimens:

- Control group (C group): Rats received distilled water only throughout the experimental period.
- Chromium group (Cr group): Animals were administered potassium dichromate (K_2_Cr_2_O_7_), a hexavalent chromium compound, dissolved in distilled water for a period of three months.
- Chromium + *Hypericum perforatum* group (CrH group): Animals received a combination of CrVI and a 2.5% aqueous extract of *Hypericum perforatum* in distilled water for three months.
- Chromium withdrawal group (Cr2 group): Animals were administered CrVI in distilled water for three months, followed by a one-month recovery period during which only distilled water was provided.
- Chromium + *Hypericum perforatum* withdrawal group (CrH2 group): Animals received CrVI in distilled water for three months, followed by one month of treatment with a 2.5% aqueous extract of *Hypericum perforatum* in distilled water.

At the end of the exposure period, all animals were euthanised by an overdose of ketamine (Ketamine 10%, CP-Pharma, Burgdorf, Germany) in combination with xylazine (Narcoxyl, Intervet International, Boxmeer, The Netherlands). Following euthanasia, the pancreas was collected for further histological and biochemical analyses.

All procedures involving animal handling, treatment, and euthanasia were carried out in strict accordance with the European Directive 2010/63/EU on the protection of animals used for scientific purposes and with the NRC Guidelines [116,117].

### 4.1. HPLC Analysis

The reference standard St John’s wort dry extract HRS 3 (code Y0001050, batch 3.0, id 00db1j from EDQM, Strasbourg, France) and sample solutions were filtered into HPLC vials and placed in position in a Jasco HPLC chromatographic system equipped with an autosampler, UV–Vis detector, and a Merck LiChrospher RP-18 endcapped 250 × 4.6 mm, 5 μm column. The column temperature was set to 35 °C and the injection volume to 10 µL. The elution was performed using a two-mobile-phase gradient. Mobile phase A contained H_3_PO_4_:water (3:1000, *v*/*v*), while mobile phase B contained H_3_PO_4_:acetonitril (3:1000, *v*/*v*). The detailed mobile phase gradient (based on European Pharmacopeia 7.0 protocol [118]) is described in Table 3 with a flow rate from 0.8 to 1.2 mL/min. The wavelengths on the UV–Visible detector were set to 360 nm.

### 4.2. Histological Assessment

Pancreas samples were processed using the standard paraffin-embedding technique. The samples were fixed in Bouin–Hollande solution for 18 h, after which they were thoroughly rinsed in distilled water. The samples were then dehydrated using a graded ethanol series (30% to 100%), cleared in xylene and embedded in paraffin wax (paraffin pastilles for histology, Merck, Darmstadt, Germany).

The paraffin blocks were then sectioned at 5 µm thickness using a microtome (Cut 4062, Slee, Mainz, Germany). The sections were mounted onto glass slides and subjected to routine histological staining. Haematoxylin and Eosin (H.E.) staining was applied to evaluate general tissue architecture.

The stained slides were examined under a light microscope (Olympus CX41RF, Olympus Life and Material Science, Southend-on-Sea, UK), which was equipped with Olympus DP23 Digital Microscope Camera and cellSENS Microscope Imaging Software (CS-EN-V4.3-EL cellSENS ENTRY version 4.3, UK).

Pancreatic oedema was assessed on H&E-stained sections using a semi-quantitative score from 0 to 3: 0 = no oedema, 1 = interlobular oedema, 2 = interlobular and moderate intralobular oedema, and 3 = severe interlobular and intralobular oedema [119].

### 4.3. Quantitative Real-Time PCR (qRT-PCR)

To investigate apoptosis-related molecular responses, the expression of *Bax* and *Bcl2* genes was quantified, with the housekeeping gene *GAPDH* serving as an internal reference. Pancreatic tissue fragments (≈50 mg) were excised, immediately immersed in RNAlater™ Stabilisation Solution (Thermo Fisher Scientific, Waltham, MA, USA), and stored at −80 °C until processing. Total RNA was extracted using the SV Total RNA Isolation System (Promega, Madison, WI, USA), following the manufacturer’s guidelines. RNA concentration and purity were assessed spectrophotometrically on a NanoDrop™ 8000 (Thermo Fisher Scientific, USA), and only samples with A260/A280 values between 1.8 and 2.0 were used for downstream analyses.

First-strand cDNA synthesis was performed from 2 µg of total RNA using the RevertAid First Strand cDNA Synthesis Kit (Thermo Fisher Scientific, Vilnius, Lithuania) according to the supplier’s protocol. For subsequent amplification, 150 ng of cDNA template was subjected to real-time PCR using the GoTaq^®^ qPCR Master Mix (Promega, Madison, WI, USA) on a 7500 Real-Time PCR System (Applied Biosystems, Foster City, CA, USA).

The primers used in this study (Table 4) were selected from previously published, validated primer sets widely used in rat experimental models and available in public databases. Amplification specificity was assessed during each qRT-PCR run by melt-curve analysis, which consistently demonstrated a single, sharp peak for each target gene across all experimental groups, indicating specific amplification without nonspecific products or primer–dimer formation.

No-template controls (NTC) and no reverse-transcription controls (–RT) were included in all assays and showed no detectable amplification.

All reactions were carried out in triplicate to ensure reproducibility. Relative quantification of target genes was calculated using the 2^−ΔΔCt^ method, and results were expressed as fold change relative to the control group.

### 4.4. Blood Glucose Levels Assessment

At the end of the experimental period, blood glucose levels were measured using a portable glucometer (ACCU-CHEK Active, Model GC, Roche Diagnostics, Mannheim, Germany) with the manufacturer’s test strips. Blood samples were collected from the tail vein. A drop of whole blood was immediately applied to the test strip inserted into the glucometer, which automatically recorded the glycaemia value.

### 4.5. Statistical Analysis

Data were analysed using Stata software (version 13). Prior to statistical testing, the assumptions required for one-way ANOVA were verified. The normality of the residuals was assessed using the Shapiro–Wilk test and by inspection of Q–Q plots, while homogeneity of variances was evaluated using Levene’s test.

When assumptions were satisfied, differences between experimental groups were analysed using one-way ANOVA followed by Tukey’s Honestly Significant Difference (HSD) post hoc test for multiple pairwise comparisons. Results are expressed as mean ± standard deviation (SD). A probability value of *p* < 0.05 was considered statistically significant.

## 5. Conclusions

The results of this study confirm that exposure to hexavalent chromium affects the endocrine pancreas, causing structural and molecular changes that disrupt carbohydrate metabolism. At the same time, the administration of aqueous St. John’s wort extract has been shown to diminish these harmful effects, highlighting its antioxidant properties and its potential use as a protective agent against CrVI-induced oxidative stress. Nevertheless, St. John’s wort remains a rich source of bioactive compounds, and its therapeutic potential for the prevention and treatment of various diseases remains largely unexplored.

## Figures and Tables

**Table 1:** Pancreatic oedema scores in rats following CrVI exposure and *Hypericum perforatum* extract intervention.

Groups/Oedema Score	0	1	2	3
C group	x			
Cr group				x
CrH group		x		
Cr2 group				x
CrH2 group	x			

The numbers represent the predominant histological score observed in each experimental group.

**Table 2:** Presentation of the experimental protocol.

	Months			
Groups	1	2	3	4
C group	Distilled water only			
Cr group	CrVI			-
CrH group	CrVI + *Hypericum perforatum* extract			-
Cr2 group	CrVI			Water
CrH2 group	CrVI			*Hypericum perforatum* extract

**Table 3:** The detailed mobile phase gradient used [118].

Time [min]	Mobile Phase A [*v*/*v*]	Mobile Phase B [*v*/*v*]	Flow Rate [mL/min]
0–8	82	18	0.8
8–18	82 → 47	18 → 53	0.8
18–18.1	47 → 3	53 → 97	0.8
18.1 → 19	3	97	0.8 → 1.2
19–29	3	97	1.2
29–30	3–82	97 → 18	1.2

**Table 4:** Primer sequences used for qRT-PCR analysis (5′–3′).

Gene	Forward Primer (5′–3′)	Forward Primer (5′–3′)
*GAPDH*	ATGGAGAAGGCTGGGGCTCACCT	AGCCCTTCCACGATGCCAAAGTTGT
*Bax*	CCAGGACGCATCCACCAAGAAGC	TGCCACACGGAAGAAGACCTCTCG
*Bcl2*	GGATGACTTCTCTCGTCGCTACCGT	ATCCCTGAAGAGTTCCTCCACCAC

## Data Availability

Data are contained within the article.

## Abbreviations

The following abbreviations are used in this manuscript:

µM	Micromoles per liter
AMPK	AMP-activated protein kinases
C	Control
Cr	Chromium
Cr2	Chromium withdrawal group
CrH	Chromium and *Hypericum perforatum*
CrH2	Chromium and *Hypericum perforatum* withdrawal group
CrIII	Trivalent chromium
CrVI	Hexavalent chromium
DNA	Deoxyribonucleic acid
H.E.	Haematoxylin and Eosin
*H. perforatum*	*Hypericum perforatum*
Kg b.w.	Kilogram body weight
LOAEL	Lowest observed adverse effect level
mg	Milligrams
qRT-PCR	Quantitative Real-Time PCR
RNA	Ribonucleic acid
ROS	Reactive oxygen species

## References

[1] Bagchi D., Stohs S.J., Downs B.W., Bagchi M., Preuss H.G. (2002). Cytotoxicity and Oxidative Mechanisms of Different Forms of Chromium. Toxicology.

[2] Wu Y., Lin J., Wang T., Lin T., Yen M., Liu Y., Wu P., Chen F., Shih Y., Yeh I. (2019). Hexavalent Chromium Intoxication Induces Intrinsic and Extrinsic Apoptosis in Human Renal Cells. Mol. Med. Rep..

[3] Codd R., Dillon C.T., Levina A., Lay P.A. (2001). Studies on the Genotoxicity of Chromium: From the Test Tube to the Cell. Coord. Chem. Rev..

[4] (2024). IRIS Toxicological Review of Hexavalent Chromium.

[5] OEHHA Chromium-Hexavalent. https://oehha.ca.gov/chemicals/chromium-hexavalent.

[6] Hossini H., Shafie B., Niri A.D., Nazari M., Esfahlan A.J., Ahmadpour M., Nazmara Z., Ahmadimanesh M., Makhdoumi P., Mirzaei N. (2022). A Comprehensive Review on Human Health Effects of Chromium: Insights on Induced Toxicity. Environ. Sci. Pollut. Res. Int..

[7] Quinteros F.A., Poliandri A.H.B., Machiavelli L.I., Cabilla J.P., Duvilanski B.H. (2007). In Vivo and in Vitro Effects of Chromium VI on Anterior Pituitary Hormone Release and Cell Viability. Toxicol. Appl. Pharmacol..

[8] Nudler S.I., Quinteros F.A., Miler E.A., Cabilla J.P., Ronchetti S.A., Duvilanski B.H. (2009). Chromium VI Administration Induces Oxidative Stress in Hypothalamus and Anterior Pituitary Gland from Male Rats. Toxicol. Lett..

[9] Savici J., Boldura O.-M., Balta C., Muselin F., Mederle N., Cristina R.T., Brezovan D. (2023). Effects of Aronia Melanocarpa and *Hypericum perforatum* Aqueous Extracts on Hexavalent Chromium Induced Toxicity in Rat’s Thyrocytes. J. Trace Elem. Med. Biol..

[10] Qureshi I.Z., Mahmood T. (2010). Prospective Role of Ascorbic Acid (Vitamin C) in Attenuating Hexavalent Chromium-Induced Functional and Cellular Damage in Rat Thyroid. Toxicol. Ind. Health.

[11] Fedala A., Adjroud O., Abid-Essefi S., Timoumi R. (2021). Protective Effects of Selenium and Zinc against Potassium Dichromate-Induced Thyroid Disruption, Oxidative Stress, and DNA Damage in Pregnant Wistar Rats. Environ. Sci. Pollut. Res. Int..

[12] Hassanin K.M.A., Abd El-Kawi S.H., Hashem K.S. (2013). The Prospective Protective Effect of Selenium Nanoparticles against Chromium-Induced Oxidative and Cellular Damage in Rat Thyroid. Int. J. Nanomed..

[13] ElBakry R.H., Tawfik S.M. (2014). Histological Study of the Effect of Potassium Dichromate on the Thyroid Follicular Cells of Adult Male Albino Rat and the Possible Protective Role of Ascorbic Acid (Vitamin C). J. Microsc. Ultrastruct..

[14] Mahmood T., Qureshi I.Z., Iqbal M.J. (2010). Histopathological and Biochemical Changes in Rat Thyroid Following Acute Exposure to Hexavalent Chromium. Histol. Histopathol..

[15] Savici J., Boldura O.M., Balta C., Brezovan D., Muselin F., Mircu C., Borza C., Motoc M., Tulcan C. (2017). Protective Effect of *Hypericum perforatum* L. Extract on Hexavalent Chromium Induced Toxicity in Rat Adrenal Gland. Rev. Chim..

[16] Solis-Heredia M.J., Quintanilla-Vega B., Sierra-Santoyo A., Hernández J.M., Brambila E., Cebrián M.E., Albores A. (1999). Chromium Increases Pancreatic Metallothionein in the Rat. Toxicology.

[17] El-Saad A.M.A., Abdel-Moneim A.M., Abdel-Karim H.M. (2010). N-Acetylcysteine an Allium Plant Compound Protects against Chromium (VI) Induced Oxidant Stress and Ultrastructural Changes of Pancreatic Beta-Cells in Rats. J. Med. Plants Res..

[18] Guria S., Chakraborty B., Banerjee M. (2016). Chromiu(VI) Induced Histological Changes of Pancreatic Islets and Liver: A Prleiminary Study of Metal Induced Diabetes Melitus. Experiment.

[19] Singh V., Singh N., Verma M., Kamal R., Tiwari R., Sanjay Chivate M., Rai S.N., Kumar A., Singh A., Singh M.P. (2022). Hexavalent-Chromium-Induced Oxidative Stress and the Protective Role of Antioxidants against Cellular Toxicity. Antioxidants.

[20] Chiu A., Shi X.L., Lee W.K.P., Hill R., Wakeman T.P., Katz A., Xu B., Dalal N.S., Robertson J.D., Chen C. (2010). Review of Chromium (VI) Apoptosis, Cell-Cycle-Arrest, and Carcinogenesis. J. Environ. Sci. Health C.

[21] Elmore S. (2007). Apoptosis: A Review of Programmed Cell Death. Toxicol. Pathol..

[22] Zhang X., Wang Y., Chen M., Zeng M. (2021). Hexavalent Chromium-Induced Apoptosis in Hep3B Cells Is Accompanied by Calcium Overload, Mitochondrial Damage, and AIF Translocation. Ecotoxicol. Environ. Saf..

[23] Bagchi D., Bagchi M., Stohs S.J. (2001). Chromium (VI)-Induced Oxidative Stress, Apoptotic Cell Death and Modulation of P53 Tumor Suppressor Gene. Mol. Cell. Biochem..

[24] Błońska-Sikora E., Zielińska A., Dobros N., Paradowska K., Michalak M. (2025). Polyphenol and Flavonoid Content and Antioxidant Activity of *Hypericum perforatum* L. (St. John’s Wort) Extracts for Potential Pharmaceutical and Cosmetic Applications. Appl. Sci..

[25] Mizuno H., Taketomi A., Nakabayashi T. (2018). Potentially Beneficial Effects of St. John’s Wort (Hypericum Perforatum) in Patients with Metabolic Syndrome. OBM Integr. Complement. Med..

[26] Alahmad A., Alghoraibi I., Zein R., Kraft S., Dräger G., Walter J.-G., Scheper T. (2022). Identification of Major Constituents of *Hypericum perforatum* L. Extracts in Syria by Development of a Rapid, Simple, and Reproducible HPLC-ESI-Q-TOF MS Analysis and Their Antioxidant Activities. ACS Omega.

[27] El-Sherbiny D.A., Khalifa A.E., Attia A.S., Eldenshary E.E.-D.S. (2003). *Hypericum perforatum* Extract Demonstrates Antioxidant Properties against Elevated Rat Brain Oxidative Status Induced by Amnestic Dose of Scopolamine. Pharmacol. Biochem. Behav..

[28] Sánchez-Reus M.I., Gómez del Rio M.A., Iglesias I., Elorza M., Slowing K., Benedí J. (2007). Standardized *Hypericum perforatum* Reduces Oxidative Stress and Increases Gene Expression of Antioxidant Enzymes on Rotenone-Exposed Rats. Neuropharmacology.

[29] Bayramoglu G., Bayramoglu A., Engur S., Senturk H., Ozturk N., Colak S. (2014). The Hepatoprotective Effects of *Hypericum perforatum* L. on Hepatic Ischemia/Reperfusion Injury in Rats. Cytotechnology.

[30] Zou Y., Lu Y., Wei D. (2004). Antioxidant Activity of a Flavonoid-Rich Extract of *Hypericum perforatum* L. in Vitro. J. Agric. Food Chem..

[31] Silva B.A., Ferreres F., Malva J.O., Dias A.C.P. (2005). Phytochemical and Antioxidant Characterization of *Hypericum perforatum* Alcoholic Extracts. Food Chem..

[32] Kakouri E., Trigas P., Daferera D., Skotti E., Tarantilis P.A., Kanakis C. (2023). Chemical Characterization and Antioxidant Activity of Nine Hypericum Species from Greece. Antioxidants.

[33] Benedí J., Arroyo R., Romero C., Martín-Aragón S., Villar A.M. (2004). Antioxidant Properties and Protective Effects of a Standardized Extract of *Hypericum perforatum* on Hydrogen Peroxide-Induced Oxidative Damage in PC12 Cells. Life Sci..

[34] Novelli M., Menegazzi M., Beffy P., Porozov S., Gregorelli A., Giacopelli D., De Tata V., Masiello P. (2016). St. John’s Wort Extract and Hyperforin Inhibit Multiple Phosphorylation Steps of Cytokine Signaling and Prevent Inflammatory and Apoptotic Gene Induction in Pancreatic β Cells. Int. J. Biochem. Cell Biol..

[35] Novelli M., Beffy P., Menegazzi M., De Tata V., Martino L., Sgarbossa A., Porozov S., Pippa A., Masini M., Marchetti P. (2014). St. John’s Wort Extract and Hyperforin Protect Rat and Human Pancreatic Islets against Cytokine Toxicity. Acta Diabetol..

[36] Menegazzi M., Novelli M., Beffy P., D’Aleo V., Tedeschi E., Lupi R., Zoratti E., Marchetti P., Suzuki H., Masiello P. (2008). Protective Effects of St. John’s Wort Extract and Its Component Hyperforin against Cytokine-Induced Cytotoxicity in a Pancreatic β-Cell Line. Int. J. Biochem. Cell Biol..

[37] Genovese T., Mazzon E., Di Paola R., Muià C., Crisafulli C., Menegazzi M., Malleo G., Suzuki H., Cuzzocrea S. (2006). *Hypericum perforatum* Attenuates the Development of Cerulein-Induced Acute Pancreatitis in Mice. Shock.

[38] Yoshida M., Hatakeyama E., Hosomi R., Kanda S., Nishiyama T., Fukunaga K. (2010). Tissue Accumulation and Urinary Excretion of Chromium in Rats Fed Diets Containing Graded Levels of Chromium Chloride or Chromium Picolinate. J. Toxicol. Sci..

[39] Salama A., Hegazy R., Hassan A. (2016). Intranasal Chromium Induces Acute Brain and Lung Injuries in Rats: Assessment of Different Potential Hazardous Effects of Environmental and Occupational Exposure to Chromium and Introduction of a Novel Pharmacological and Toxicological Animal Model. PLoS ONE.

[40] Pereira S.C., Oliveira P.F., Oliveira S.R., Pereira M.D.L., Alves M.G. (2021). Impact of Environmental and Lifestyle Use of Chromium on Male Fertility: Focus on Antioxidant Activity and Oxidative Stress. Antioxidants.

[41] Li X., He S., Zhou J., Yu X., Li L., Liu Y., Li W. (2021). Cr (VI) Induces Abnormalities in Glucose and Lipid Metabolism through ROS/Nrf2 Signaling. Ecotoxicol. Environ. Saf..

[42] Mishra S., Bharagava R.N. (2016). Toxic and Genotoxic Effects of Hexavalent Chromium in Environment and Its Bioremediation Strategies. J. Environ. Sci. Health C Environ. Carcinog. Ecotoxicol. Rev..

[43] Wakeel A., Xu M., Gan Y. (2020). Chromium-Induced Reactive Oxygen Species Accumulation by Altering the Enzymatic Antioxidant System and Associated Cytotoxic, Genotoxic, Ultrastructural, and Photosynthetic Changes in Plants. Int. J. Mol. Sci..

[44] Kumar M.S., Praveenkumar R., Ilavarasi A., Rajeshwari K., Thajuddin N. (2013). Biochemical Changes of Fresh Water Cyanobacteria Dolichospermum Flos-Aquae NTMS07 to Chromium-Induced Stress with Special Reference to Antioxidant Enzymes and Cellular Fatty Acids. Bull. Environ. Contam. Toxicol..

[45] Singh V., Singh J., Mishra V. (2021). Development of a Cost-Effective, Recyclable and Viable Metal Ion Doped Adsorbent for Simultaneous Adsorption and Reduction of Toxic Cr (VI) Ions. J. Environ. Chem. Eng..

[46] Joutey N.T., Sayel H., Bahafid W., El Ghachtouli N., Whitacre D.M. (2015). Mechanisms of Hexavalent Chromium Resistance and Removal by Microorganisms. Reviews of Environmental Contamination and Toxicology.

[47] Arulselvan P., Subramanian S.P. (2007). Beneficial Effects of Murraya Koenigii Leaves on Antioxidant Defense System and Ultra Structural Changes of Pancreatic Beta-Cells in Experimental Diabetes in Rats. Chem. Biol. Interact..

[48] Zhou J., Zhou S., Tang J., Zhang K., Guang L., Huang Y., Xu Y., Ying Y., Zhang L., Li D. (2009). Protective Effect of Berberine on Beta Cells in Streptozotocin- and High-Carbohydrate/High-Fat Diet-Induced Diabetic Rats. Eur. J. Pharmacol..

[49] García-Niño W.R., Zatarain-Barrón Z.L., Hernández-Pando R., Vega-García C.C., Tapia E., Pedraza-Chaverri J. (2015). Oxidative Stress Markers and Histological Analysis in Diverse Organs from Rats Treated with a Hepatotoxic Dose of Cr(VI): Effect of Curcumin. Biol. Trace Elem. Res..

[50] Han W., Song Y., Rocha M., Shi Y. (2023). Ischemic Brain Edema: Emerging Cellular Mechanisms and Therapeutic Approaches. Neurobiol. Dis..

[51] Tran Q. (2000). Calcium Signalling in Endothelial Cells. Cardiovasc. Res..

[52] Núñez O., Fernández-Navarro P., Martín-Méndez I., Bel-Lan A., Locutura J.F., López-Abente G. (2016). Arsenic and Chromium Topsoil Levels and Cancer Mortality in Spain. Environ. Sci. Pollut. Res. Int..

[53] El-Atta H.M.A., El-Bakary A.A., Attia A.M., Lotfy A., Khater S.S., Elsamanoudy A.Z., Abdalla H.A. (2014). DNA Fragmentation, Caspase 3 and Prostate-Specific Antigen Genes Expression Induced by Arsenic, Cadmium, and Chromium on Nontumorigenic Human Prostate Cells. Biol. Trace Elem. Res..

[54] Xiao F., Li Y., Dai L., Deng Y., Zou Y., Li P., Yang Y., Zhong C. (2012). Hexavalent Chromium Targets Mitochondrial Respiratory Chain Complex I to Induce Reactive Oxygen Species-Dependent Caspase-3 Activation in L-02 Hepatocytes. Int. J. Mol. Med..

[55] Molina-Jijón E., Tapia E., Zazueta C., El Hafidi M., Zatarain-Barrón Z.L., Hernández-Pando R., Medina-Campos O.N., Zarco-Márquez G., Torres I., Pedraza-Chaverri J. (2011). Curcumin Prevents Cr(VI)-Induced Renal Oxidant Damage by a Mitochondrial Pathway. Free Radic. Biol. Med..

[56] Banu S.K., Stanley J.A., Sivakumar K.K., Arosh J.A., Taylor R.J., Burghardt R.C. (2017). Chromium VI—Induced Developmental Toxicity of Placenta Is Mediated through Spatiotemporal Dysregulation of Cell Survival and Apoptotic Proteins. Reprod. Toxicol..

[57] Banu S.K., Stanley J.A., Sivakumar K.K., Arosh J.A., Burghardt R.C. (2016). Resveratrol Protects the Ovary against Chromium-Toxicity by Enhancing Endogenous Antioxidant Enzymes and Inhibiting Metabolic Clearance of Estradiol. Toxicol. Appl. Pharmacol..

[58] Marouani N., Tebourbi O., Mokni M., Yacoubi M.T., Sakly M., Benkhalifa M., Rhouma K.B. (2015). Hexavalent Chromium-Induced Apoptosis in Rat Uterus: Involvement of Oxidative Stress. Arch. Environ. Occup. Health.

[59] He L., He T., Farrar S., Ji L., Liu T., Ma X. (2017). Antioxidants Maintain Cellular Redox Homeostasis by Elimination of Reactive Oxygen Species. Cell. Physiol. Biochem..

[60] Zeliou K., Kontaxis N.I., Margianni E., Petrou C., Lamari F.N. (2017). Optimized and Validated HPLC Analysis of St. John’s Wort Extract and Final Products by Simultaneous Determination of Major Ingredients. J. Chromatogr. Sci..

[61] Zvezdanović J., Petrović S., Savić S., Cvetković D., Stanojević L., Stanojević J., Lazarević A. (2021). Phenolics and Mineral Content in St. John’s Wort Infusions from Serbia Origin: An HPLC and ICP-OES Study. Chem. Pap..

[62] Kilibarda S., Jović M.D., Milinčić D.D., Vuković S., Trifković J.Đ., Pešić M.B., Kostić A.Ž (2025). Phytochemical Profile and Biological Activities of Rtanj’s *Hypericum Perforatum* Infusion Tea and Methanolic Extracts: Insights from LC-MS/MS and HPTLC–Bioautography. Plants.

[63] Liu Z., Tao X., Zhang C., Lu Y., Wei D. (2005). Protective Effects of Hyperoside (Quercetin-3-o-Galactoside) to PC12 Cells against Cytotoxicity Induced by Hydrogen Peroxide and Tert-Butyl Hydroperoxide. Biomed. Pharmacother..

[64] Silva B.A., Malva J.O., Dias A.C.P. (2008). St. John’s Wort (*Hypericum perforatum*) Extracts and Isolated Phenolic Compounds Are Effective Antioxidants in Several in Vitro Models of Oxidative Stress. Food Chem..

[65] Luo L., Sun Q., Mao Y.Y., Lu Y.H., Tan R.X. (2004). Inhibitory Effects of Flavonoids from *Hypericum perforatum* on Nitric Oxide Synthase. J. Ethnopharmacol..

[66] Zeng K.-W., Wang X.-M., Ko H., Kwon H.C., Cha J.W., Yang H.O. (2011). Hyperoside Protects Primary Rat Cortical Neurons from Neurotoxicity Induced by Amyloid β-Protein via the PI3K/Akt/Bad/Bcl(XL)-Regulated Mitochondrial Apoptotic Pathway. Eur. J. Pharmacol..

[67] Ramos A.A., Lima C.F., Pereira M.L., Fernandes-Ferreira M., Pereira-Wilson C. (2008). Antigenotoxic Effects of Quercetin, Rutin and Ursolic Acid on HepG2 Cells: Evaluation by the Comet Assay. Toxicol. Lett..

[68] Yang J., Guo J., Yuan J. (2008). In Vitro Antioxidant Properties of Rutin. LWT-Food Sci. Technol..

[69] Song K., Kim S., Na J.-Y., Park J.-H., Kim J.-K., Kim J.-H., Kwon J. (2014). Rutin Attenuates Ethanol-Induced Neurotoxicity in Hippocampal Neuronal Cells by Increasing Aldehyde Dehydrogenase 2. Food Chem. Toxicol..

[70] Alía M., Mateos R., Ramos S., Lecumberri E., Bravo L., Goya L. (2006). Influence of Quercetin and Rutin on Growth and Antioxidant Defense System of a Human Hepatoma Cell Line (HepG2). Eur. J. Nutr..

[71] Khan M.M., Ahmad A., Ishrat T., Khuwaja G., Srivastawa P., Khan M.B., Raza S.S., Javed H., Vaibhav K., Khan A. (2009). Rutin Protects the Neural Damage Induced by Transient Focal Ischemia in Rats. Brain Res..

[72] Heo H.J., Lee C.Y. (2004). Protective Effects of Quercetin and Vitamin C against Oxidative Stress-Induced Neurodegeneration. J. Agric. Food Chem..

[73] Suematsu N., Hosoda M., Fujimori K. (2011). Protective Effects of Quercetin against Hydrogen Peroxide-Induced Apoptosis in Human Neuronal SH-SY5Y Cells. Neurosci. Lett..

[74] Silva B., Oliveira P.J., Dias A., Malva J.O. (2008). Quercetin, Kaempferol and Biapigenin from *Hypericum perforatum* Are Neuroprotective against Excitotoxic Insults. Neurotox. Res..

[75] Boots A.W., Haenen G.R.M.M., Bast A. (2008). Health Effects of Quercetin: From Antioxidant to Nutraceutical. Eur. J. Pharmacol..

[76] Dok-Go H., Lee K.H., Kim H.J., Lee E.H., Lee J., Song Y.S., Lee Y.-H., Jin C., Lee Y.S., Cho J. (2003). Neuroprotective Effects of Antioxidative Flavonoids, Quercetin, (+)-Dihydroquercetin and Quercetin 3-Methyl Ether, Isolated from *Opuntia ficus-indica* Var. *Saboten*. Brain Res..

[77] Hollman P.C., de Vries J.H., van Leeuwen S.D., Mengelers M.J., Katan M.B. (1995). Absorption of Dietary Quercetin Glycosides and Quercetin in Healthy Ileostomy Volunteers. Am. J. Clin. Nutr..

[78] Wagner C., Fachinetto R., Dalla Corte C.L., Brito V.B., Severo D., de Oliveira Costa Dias G., Morel A.F., Nogueira C.W., Rocha J.B.T. (2006). Quercitrin, a Glycoside Form of Quercetin, Prevents Lipid Peroxidation in Vitro. Brain Res..

[79] Bergqvist F., Morgenstern R., Jakobsson P.-J. (2020). A Review on mPGES-1 Inhibitors: From Preclinical Studies to Clinical Applications. Prostaglandins Other Lipid Mediat..

[80] Novelli M., Masiello P., Beffy P., Menegazzi M. (2020). Protective Role of St. John’s Wort and Its Components Hyperforin and Hypericin against Diabetes through Inhibition of Inflammatory Signaling: Evidence from In Vitro and In Vivo Studies. Int. J. Mol. Sci..

[81] McLaughlin T., Liu L.-F., Lamendola C., Shen L., Morton J., Rivas H., Winer D., Tolentino L., Choi O., Zhang H. (2014). T-Cell Profile in Adipose Tissue Is Associated with Insulin Resistance and Systemic Inflammation in Humans. Arter. Thromb. Vasc. Biol..

[82] Abd El Motteleb D.M., Abd El Aleem D.I. (2017). Renoprotective Effect of *Hypericum perforatum* against Diabetic Nephropathy in Rats: Insights in the Underlying Mechanisms. Clin. Exp. Pharmacol. Physiol..

[83] Nadipelly J. (2017). Molecular Mechanisms Involved in Inflammatory Cascade: A Review. Texila Int. J. Basic Med. Sci..

[84] Tang F., Yan H.-L., Wang L.-X., Xu J.-F., Peng C., Ao H., Tan Y.-Z. (2021). Review of Natural Resources with Vasodilation: Traditional Medicinal Plants, Natural Products, and Their Mechanism and Clinical Efficacy. Front. Pharmacol..

[85] Jang J.H., Surh Y.J. (2001). Protective Effects of Resveratrol on Hydrogen Peroxide-Induced Apoptosis in Rat Pheochromocytoma (PC12) Cells. Mutat. Res..

[86] Jang M.-H., Lee T.-H., Shin M.-C., Bahn G.-H., Kim J.-W., Shin D.-H., Kim E.-H., Kim C.-J. (2002). Protective Effect of *Hypericum perforatum* Linn (St. John’s Wort) against Hydrogen Peroxide-Induced Apoptosis on Human Neuroblastoma Cells. Neurosci. Lett..

[87] Tripathi Y.B., Pandey E. (1999). Role of Alcoholic Extract of Shoot of *Hypericum perforatum* Linn on Lipid Peroxidation and Various Species of Free Radicals in Rats. Indian J. Exp. Biol..

[88] Kim E., Na K.J. (1991). Effect of Sodium Dichromate on Carbohydrate Metabolism. Toxicol. Appl. Pharmacol..

[89] Das Gupta A., Dhara P.C., Dhundasi S.A., Das K.K. (2009). Effect of Garlic (Allium Sativum) on Nickel II or Chromium VI Induced Alterations of Glucose Homeostasis and Hepatic Antioxidant Status under Sub-Chronic Exposure Conditions. J. Basic Clin. Physiol. Pharmacol..

[90] El-Demerdash F., Yousef M., Elaswad F. (2006). Biochemical Study on the Protective Role of Folic Acid in Rabbits Treated with Chromium (VI). J. Environ. Sci. Health B.

[91] Cunha D.A., Cito M., Grieco F.A., Cosentino C., Danilova T., Ladrière L., Lindahl M., Domanskyi A., Bugliani M., Marchetti P. (2017). Pancreatic β-Cell Protection from Inflammatory Stress by the Endoplasmic Reticulum Proteins Thrombospondin 1 and Mesencephalic Astrocyte-Derived Neutrotrophic Factor (MANF). J. Biol. Chem..

[92] Chan J.Y., Luzuriaga J., Maxwell E.L., West P.K., Bensellam M., Laybutt D.R. (2015). The Balance between Adaptive and Apoptotic Unfolded Protein Responses Regulates β-Cell Death under ER Stress Conditions through XBP1, CHOP and JNK. Mol. Cell. Endocrinol..

[93] Al-Ghamdi B.A., Al-Shamrani J.M., El-Shehawi A.M., Al-Johani I., Al-Otaibi B.G. (2022). Role of Mitochondrial DNA in Diabetes Mellitus Type I and Type II. Saudi J. Biol. Sci..

[94] Kim J., Yang G., Kim Y., Kim J., Ha J. (2016). AMPK Activators: Mechanisms of Action and Physiological Activities. Exp. Mol. Med..

[95] Ren Y., Shen H.-M. (2019). Critical Role of AMPK in Redox Regulation under Glucose Starvation. Redox Biol..

[96] Chung M.-Y., Choi H.-K., Hwang J.-T. (2021). AMPK Activity: A Primary Target for Diabetes Prevention with Therapeutic Phytochemicals. Nutrients.

[97] Meng S., Cao J., Feng Q., Peng J., Hu Y. (2013). Roles of Chlorogenic Acid on Regulating Glucose and Lipids Metabolism: A Review. Evid. Based Complement. Altern. Med..

[98] Liang C., Li Y., Bai M., Huang Y., Yang H., Liu L., Wang S., Yu C., Song Z., Bao Y. (2020). Hypericin Attenuates Nonalcoholic Fatty Liver Disease and Abnormal Lipid Metabolism via the PKA-Mediated AMPK Signaling Pathway In Vitro and In Vivo. Pharmacol. Res..

[99] Chen L., Shen T., Zhang C.P., Xu B.L., Qiu Y.Y., Xie X.Y., Wang Q., Lei T. (2020). Quercetin and Isoquercitrin Inhibiting Hepatic Gluconeogenesis Through LKB1-AMPKα Pathway. Acta Endocrinol..

[100] Husain G.M., Singh P.N., Kumar V. (2009). Beneficial Effects of a Standardized *Hypericum perforatum* Extract in Rats with Experimentally Induced Hyperglycemia. Drug Discov. Ther..

[101] Arokiyaraj S., Balamurugan R., Augustian P. (2011). Antihyperglycemic Effect of *Hypericum perforatum* Ethyl Acetate Extract on Streptozotocin-Induced Diabetic Rats. Asian Pac. J. Trop. Biomed..

[102] Can Ö.D., Öztürk Y., Öztürk N., Sagratini G., Ricciutelli M., Vittori S., Maggi F. (2011). Effects of Treatment with St. John’s Wort on Blood Glucose Levels and Pain Perceptions of Streptozotocin-Diabetic Rats. Fitoterapia.

[103] Moghadam M.G., Ansari I., Roghani M., Ghanem A., Mehdizade N. (2017). The Effect of Oral Administration of *Hypericum perforatum* on Serum Glucose and Lipids, Hepatic Enzymes and Lipid Peroxidation in Streptozotocin-Induced Diabetic Rats. Galen Med. J..

[104] Rafailovska E., Tushevski O., Gadzovska-Simic S., Dinevska-Kjovkarovska S., Miova B. (2022). *Hypericum perforatum* L. Hairy Root Extracts—Regulation of Glycemic, Metabolic, Serum Enzyme and Lipid Profile in STZ—Induced Diabetic Rats. Maced. Vet. Rev..

[105] Tian J., Tao R., Zhang X., Liu Q., He Y.-B., Su Y., Ji T., Ye F. (2015). Effect of *Hypericum perforatum* L. Extract on Insulin Resistance and Lipid Metabolic Disorder in High-Fat-Diet Induced Obese Mice. Phytother. Res..

[106] Rafailovska E., Tushevski O., Shijakova K., Simic S.G., Kjovkarovska S.D., Miova B. (2023). *Hypericum perforatum* L. Extract Exerts Insulinotropic Effects and Inhibits Gluconeogenesis in Diabetic Rats by Regulating AMPK Expression and PKCε Concentration. J. Ethnopharmacol..

[107] Yang H., Yang T., Heng C., Zhou Y., Jiang Z., Qian X., Du L., Mao S., Yin X., Lu Q. (2019). Quercetin Improves Nonalcoholic Fatty Liver by Ameliorating Inflammation, Oxidative Stress, and Lipid Metabolism in Db/Db Mice. Phytother. Res..

[108] Kamalakkannan N., Prince P.S.M. (2006). Antihyperglycaemic and Antioxidant Effect of Rutin, a Polyphenolic Flavonoid, in Streptozotocin-Induced Diabetic Wistar Rats. Basic Clin. Pharmacol. Toxicol..

[109] Verma N., Amresh G., Sahu P.K., Rao C.V., Singh A.P. (2012). Antihyperglycemic and Antihyperlipidemic Activity of Ethyl Acetate Fraction of Rhododendron Arboreum Smith Flowers in Streptozotocin Induced Diabetic Rats and Its Role in Regulating Carbohydrate Metabolism. Asian Pac. J. Trop. Biomed..

[110] Sarian M.N., Ahmed Q.U., Mat So’ad S.Z., Alhassan A.M., Murugesu S., Perumal V., Syed Mohamad S.N.A., Khatib A., Latip J. (2017). Antioxidant and Antidiabetic Effects of Flavonoids: A Structure-Activity Relationship Based Study. BioMed Res. Int..

[111] Torres-Piedra M., Ortiz-Andrade R., Villalobos-Molina R., Singh N., Medina-Franco J.L., Webster S.P., Binnie M., Navarrete-Vázquez G., Estrada-Soto S. (2010). A Comparative Study of Flavonoid Analogues on Streptozotocin-Nicotinamide Induced Diabetic Rats: Quercetin as a Potential Antidiabetic Agent Acting via 11beta-Hydroxysteroid Dehydrogenase Type 1 Inhibition. Eur. J. Med. Chem..

[112] Anastasiou I.A., Eleftheriadou I., Tentolouris A., Koliaki C., Kosta O.A., Tentolouris N. (2021). The Effect of Oxidative Stress and Antioxidant Therapies on Pancreatic β-Cell Dysfunction: Results from in Vitro and in Vivo Studies. Curr. Med. Chem..

[113] Menegazzi M., Masiello P., Novelli M. (2020). Anti-Tumor Activity of *Hypericum perforatum* L. and Hyperforin through Modulation of Inflammatory Signaling, ROS Generation and Proton Dynamics. Antioxidants.

[114] Liang C., Hao F., Yao X., Qiu Y., Liu L., Wang S., Yu C., Song Z., Bao Y., Yi J. (2019). Hypericin Maintians PDX1 Expression via the Erk Pathway and Protects Islet β-Cells against Glucotoxicity and Lipotoxicity. Int. J. Biol. Sci..

[115] Alupului A., Calinescu I., Lavric V. (2009). Ultrasonic vs. Microwave Extraction Intensification of Active Principles from Medicinal Plants. Chem. Eng. Trans..

[116] (2010). Directive 2010/63/EU of the European Parliament and of the Council of 22 September 2010 on the Protection of Animals Used for Scientific Purposes.

[117] National Research Council, Institute of Laboratory Animal Research (NRC) (2011). Guide for the Care and Use of Laboratory Animals.

[118] (2010). St. John’s Wort Dry Extract, Quantified—07/2008:1874, Corrected 6.3. European Pharmacopoeia 7.0.

[119] Konarska-Bajda K., Ceranowicz P., Cieszkowski J., Ginter G., Stempniewicz A., Gałązka K., Kuśnierz-Cabala B., Dumnicka P., Bonior J., Warzecha Z. (2023). Administration of Warfarin Inhibits the Development of Cerulein-Induced Edematous Acute Pancreatitis in Rats. Biomolecules.

